# Seasonal changes in gastric mucosal factors associated with peptic ulcer bleeding

**DOI:** 10.3892/etm.2014.2080

**Published:** 2014-11-19

**Authors:** XIAO-GANG YUAN, CHUAN XIE, JIANG CHEN, YONG XIE, KUN-HE ZHANG, NONG-HUA LU

**Affiliations:** Department of Gastroenterology, The First Affiliated Hospital of Nanchang University, Nanchang, Jiangxi 330006, P.R. China

**Keywords:** peptic ulcer bleeding, pathogenesis, climate

## Abstract

A close association has been established between climate and peptic ulcer bleeding (PUB). The incidence of PUB in cold climates is significantly higher than that in hot climates. In this study, gastric mucosal damage and its barrier function (through associated barrier factors) in extreme climate conditions were examined to investigate the pathogenesis of PUB in extreme cold climates. Gastric juice and biopsy specimens were collected from 176 patients with peptic ulcer. Conventional hematoxylin and eosin staining was used to exclude malignant ulcers. *Helicobacter pylori* infections were detected by modified Giemsa staining. pH values of the gastric juice samples were obtained on-site by precise pH dipstick readings. The protein expression levels of heat shock protein (HSP) 70, occludin, nitric oxide synthase (NOS), epidermal growth factor (EGF) and EGF receptor (EGFR) in the gastric mucosa were detected by immunohistochemistry. No significant differences were identified between the high and low bleeding risk groups in the rates of *H. pylori* infection and the pH values of the gastric juices in the extreme hot or cold climates. Furthermore, no statistically significant differences were identified in the protein expression levels of occludin, NOS, EGF and EGFR between the high and low bleeding risk groups. In the extreme cold climate, the expression of HSP70 and the mucus thickness of the gastric antrum in the high bleeding risk group were significantly lower than those in the low bleeding risk group. The protein expression levels of occludin, HSP70, NOS and EGFR in the extreme cold climate were significantly lower than those in the extreme hot climate, whereas the gastric acid secretion was significantly higher in the extreme cold climate than that in the extreme hot climate. In conclusion, low expression of HSP70 in the gastric mucosa and reduced gastric mucus thickness may play key roles in the mechanism of PUB in extreme cold climates. The significant decrease in barrier factors and increase in damage in extreme cold climates may be associated with the seasonal pattern of peptic ulcers.

## Introduction

In recent years, meteorological disasters, including droughts and severe winter weather, have occurred frequently in the world. A number of diseases exhibit seasonal patterns in their occurrence. Weather changes may effects cardiovascular, infectious, respiratory, gastrointestinal and other diseases. Extreme climates seriously affect health and have resulted in the increased morbidity of a number of diseases, including gastrointestinal bleeding (1), which exhibit a higher frequency in the winter than in the summer.

Peptic ulcer bleeding (PUB) is the most common cause of upper gastrointestinal bleeding, including gastric ulcers (GU) and duodenal ulcers(DU). Excessive acid secretion is discovered in some patients but may not the main factor in most ulcers. The major etiologic factors in chronic peptic ulcer are mainly caused by *Helicobacter pylori* (H. pylori) infection and nonsteroidal anti-inflammatory drugs (NSAIDs). Certain studies have indicated that the incidence of peptic ulcers in cold climates is significantly higher than that in hot climates (2,3). A previous study has additionally found that the incidence of PUB is inversely proportional to the temperature and correlated with the degree of temperature variation (3). Cold climate and rapid climate change may induce PUB (2,3), but little is known regarding its specific pathogenesis. Gastric mucosal damage and barrier factors were therefore examined in extreme climates to clarify the pathogenesis of PUB under extreme climate conditions.

## Patients and Methods

### Patients

The study was conducted with the approval of the Ethics Committee of the First Affiliated Hospital of Nanchang University (Nanchang, China). A total of 176 patients with active peptic ulcer with or without bleeding who were undergoing endoscopic examinations at the First Affiliated Hospital of Nanchang University during periods of extreme hot or cold climate were included in the study. The extreme hot climate period was between July and September 2009, when the average temperature was >30°C, and the extreme cold climate period was between December 2009 and February 2010, when the average temperature was <10°C. The patient demographics are summarized in Table I. Patients were eligible for inclusion in the present study if they met the following criteria: i) Age >18 years; ii) resident of Jiangxi province; and iii) presenting with active peptic ulcer. The active peptic ulcer had to be in conformity with one of the following conditions: Continuity of ≥5 mm, multiple ulcers or actively bleeding ulcer. The exclusion criteria were as follows: i) Serious organ diseases of important organs, including the heart, lung, liver, kidney and brain; ii) pregnant or lactating females; iii) patients with mental health problems and patients who are unable to provide a medical history; and iv) patients who have received treatment with proton pump inhibitors (PPIs) or other gastroprotective agents within the last two weeks. The subjects were divided into high and low bleeding risk groups according to their histories of PUB or observations of hemorrhages upon endoscopic examination.

### Specimen collection and processing

Gastric biopsies were collected from the patients with informed consent by endoscopy. Two specimens were collected each from the antrum and the gastric body. At the same time, 10 ml gastric juice was collected and the pH value of the gastric juice was measured precisely on-site. Biopsy specimens were fixed with 10% buffered formalin and embedded in paraffin. Sections (4 μm thick) were cut from the wax blocks. Conventional hematoxylin and eosin staining was used to exclude malignant ulcers. Periodic acid-Schiff staining was used to measure the gastric mucus thickness.

### Detection of Helicobacter pylori infection

*H. pylori* infection was detected using modified Giemsa staining. The paraffin-embedded sections were conventionally deparaffinized in xylene twice for 10 min, rehydrated through a graded ethanol series to distilled water and washed three times with distilled water. The sections were then stained for 30 min with 2% Giemsa dye, washed with running water, polarized twice with 95% alcohol, rapidly dehydrated twice with 100% alcohol, aired naturally and sealed with neutral gum. *H. pylori* infection was identified by the presence of *H. pylori* on the gastric antrum or body mucosa with an optical microscope (Nikon 55i; Nikon Corporation, Tokyo, Japan) under high magnification.

### Immunohistochemistry

The expression of heat shock protein (HSP) 70, occludin, nitric oxide synthase (NOS), epidermal growth factor (EGF) and EGF receptor (EGFR) proteins in the epithelial cells of the gastric mucous membrane was detected by immunohistochemistry. Slides were deparaffinized in xylene twice for 10 min and rehydrated through a graded ethanol series to distilled water, prior to incubation for 8 min with a 3% hydrogen peroxidase-methanol solution to inhibit endogenous peroxidase activity. The slides were heated in 0.01 mol/l citrate buffer (pH 6.0) in a microwave oven for 15 min on high power for antigen retrieval. The slides were then removed from the microwave oven and cooled at room temperature.

The sections were incubated at 4°C overnight in a humidified chamber with anti-occludin (Abcam, Cambridge, MA, USA), anti-EGF (Abcam), anti-EGFR (Santa Cruz Biotechnology, Inc., Santa Cruz, CA, USA), anti-HSP70 (Santa Cruz Biotechnology, Inc.) or anti-NOS (Santa Cruz Biotechnology, Inc.) antibodies, each of which was diluted to 1:400 in blocking solution. The sections were rinsed in phosphate-buffered saline (PBS) and incubated for 30 min with biotinylated secondary antibody (poly-peroxidase anti-mouse/rabbit immunoglobulin G; Zymed, San Francisco, CA, USA). The sections were then washed in PBS and incubated for 30 min at 37°C. 3,3′-Diaminobenzidine was used as the chromogen. The slides were counterstained for 3 min with hematoxylin solution. Specimens with known positive expression were used as positive controls for each lesion, whereas the primary antibody was replaced with PBS to serve as a negative control.

The intensity of protein expression was graded as follows: 0, no staining; 1, weak staining; 2, moderate staining; and 3, strong staining. The percentage of positive cells was evaluated as follows: 0, ≤5.0%; 1, 5.1–25.0%; 2, 25.1–50%; 3, 50.1–75%; and 4, >75%. The degree of positive staining was obtained by multiplying the intensity and the area of expression for classification as follows: Undetectable (-), score of 0–2; mild (+), score of 3–5; moderate (++), score of 6–8; and marked (+++), score of 9–12. The (−) and (+) classifications were considered to be negative results, whereas (++) and (+++) were considered to be positive results for protein expression.

### Statistical analysis

The SPSS 16.0 statistical software package (SPSS, Inc., Chicago, IL, USA) was used for statistical processing. Continuous data are expressed as the mean ± standard deviation and were compared by analysis of variance. Categorical variables were analyzed using the Mann-Whitney U test. All tests of significance were two-sided. A difference with a value of P<0.05 was considered statistically significant.

## Results

### Factors damaging the gastric mucosa

#### Level of gastric acid

The pH value of the gastric juice was lower in the high bleeding risk group (1.93±1.04) than that in the low bleeding risk group (2.05±1.27) in the extreme hot climate, but the difference was not statistically significant. This was consistent with the results for the extreme cold climate, in which no significant difference was identified in the pH values of the gastric juice between the high (1.00±0.81) and low (1.35±0.93) bleeding risk groups (Table II).

#### H. pylori infection

*H. pylori* predominantly exist in the mucosal surface, mucus and gland cavity. Under high magnification microscopy, *H. pylori* can be observed as spiral, comma or seagull shapes. In the extreme hot climate, the overall rate of *H. pylori* infection was 79.73% (59/74). No significant difference was identified between the high (75.68%, 28/37) and low (83.78%, 31/37) bleeding risk groups. In the extreme cold climate, the overall rate of *H. pylori* infection was 80.39% (82/102). No significant difference was identified in the rate of *H. pylori* infection between the high (86.27%, 44/51) and low (74.51%, 38/51) bleeding risk groups (Table II).

### Barrier factors of the gastric mucosa

**Figure 1:**
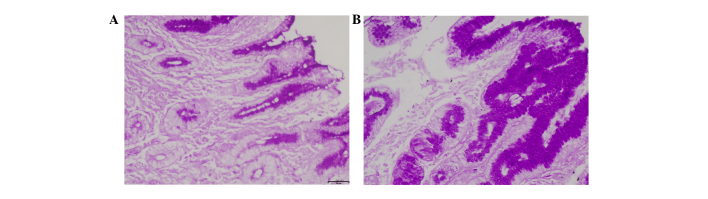
Conventional periodic acid-Schiff staining was used to detect gastric mucus thickness. (A) High and (B) low bleeding risk groups (original magnification, ×200). Mucus thickness of the gastric antrum was significantly lower in the high bleeding risk group than that in the low bleeding risk group in the extreme cold climate.

**Figure 2:**
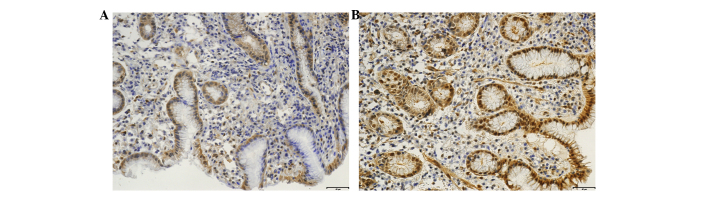
Expression of HSP70 protein in the gastric mucous membrane epithelial cells was detected by immunohistochemistry. (A) High and (B) low bleeding risk groups (brown-red indicates positive staining; original magnification, ×200). The expression of HSP70 in the high bleeding risk group was significantly lower than that in the low bleeding risk group in the extreme cold climate. HSP70, heat shock protein 70.

In the extreme hot climate, no significant differences were identified in the mucus thickness of the gastric antrum and body between the high and low bleeding risk groups. The protein expression levels of HSP70, occludin, NOS, EGF and EGFR were not significantly different between the high and low bleeding risk groups. In the extreme cold climate, the mucus thickness of the gastric antrum was significantly lower in the high bleeding risk group (4.81±1.59 μm) than that in the low bleeding risk group (5.62±1.88 μm) (Fig. 1). The expression of HSP70 in the high bleeding risk group was significantly lower than that in the low bleeding risk group (Fig. 2 and Table III). No significant differences were identified in the mucus thickness of the gastric body or the expression of the occludin, NOS, EGF and EGFR proteins between the high and low bleeding risk groups.

**Figure 3:**
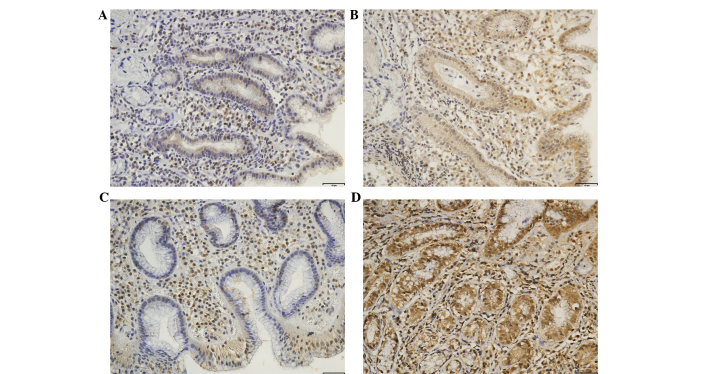
Expression of occludin and NOS proteins in the gastric mucous membrane epithelial cells was detected by immunohistochemistry. (A and C) High bleeding risk group in the extreme cold climate; (B and D) low bleeding risk group in the extreme hot climate (brown-red indicates positive staining; original magnification, ×200). The expression levels of occludin and NOS in the high bleeding risk group in the extreme cold climate were significantly lower than those in the extreme hot climate. NOS, nitric oxide synthase.

The associations between climate and factors associated with bleeding risk group were then examined. The pH of the gastric acid, the mucus thickness of the gastric antrum, the expression of the occludin and NOS proteins in the gastric antrum (Fig. 3) and the expression of the HSP70 protein in the gastric antrum and body were significantly lower in the extreme cold climate than those in the hot climate. No significant differences were identified in the mucus thickness of the gastric body and the expression of the EGF and EGFR proteins in the gastric antrum and body between the extreme cold and hot climates.

## Discussion

Acute upper gastrointestinal bleeding is a frequent and life-threatening medical emergency, and its most common etiological factor is PUB. Gastric mucosal damage and barrier factors play important roles in the development of peptic ulcers. Peptic ulcers are caused by defects in the gastro-duodenal mucosal barrier that result from epithelial cell damage, which is evoked by caustic agents, including gastric acid and pepsin (4). In general, PUB is induced by the noxious effects of damaging factors (such as acid, pepsin and *H. pylori* infection) prevailing over the barrier factors of the gastro-duodenal mucosa (5). According to Schwartz’s aphorism, ‘no acid, no ulcer’, gastric acid is the crucial aggressive factor in PUB (6).

Since the development of PPIs, the incidence of PUB has decreased (7). Furthermore, according to a previous report (8), infusion treatment with a high dose of PPI could reduce the bleeding recurrence rate, requirement for surgery, length of hospital stay and mortality rate. Peptic ulcer disease (PUD) can be effectively treated by suppressing acid, which strongly suggests that the disease is largely caused by gastric acid hypersecretion. In the present study, no significant differences were observed in the pH values of the gastric juice between the high and low bleeding risk groups in the extreme hot or cold climates; however, it was observed that the pH value in the high bleeding risk group was significantly higher in the extreme cold climate than that in the extreme hot climate. This result may explain why there was a higher incidence of peptic ulcers in the cold climate than in the hot climate.

The discovery of *H. pylori* shifted the view that PUD is simply an acid homeostasis disorder. Considerable research has established that *H. pylori* infection is closely associated with PUD (9). In the present study, no significant difference was identified in the *H. pylori* infection rate between the high or low bleeding risk groups in the extreme hot or cold climates. These results were consistent with those of Henriksson *et al* (10), which revealed no difference in the prevalence of *H. pylori* infections in different climates. Certain previous studies, however, have reported that the prevalence of *H. pylori* infection was slightly lower in patients with peptic ulcer complications than that in patients with uncomplicated ulcer disease (11,12). *H. pylori* infection and the use of nonsteroidal anti-inflammatory drugs (NSAIDs) are independent risk factors for PUB (13). Eradication of *H. pylori* can significantly reduce the risk of recurrent peptic ulcer and its complications (14). Further studies are required to determine whether *H. pylori* infection is one of the pathogenic factors for PUB in extreme climates.

Impaired defensive function of the gastro-duodenal mucosa is another important pathogenic factor of PUD. The gastro-duodenal mucosal barrier includes at least three levels. The first level of this defense system comprises the factors that are secreted into the lumen, including mucus, bicarbonates and immunoglobulins. The second level consists of the gastric epithelia. The impermeability of the gastro-duodenal epithelium makes it particularly resistant to the passive diffusion of acids or irritants. In addition, the gastro-duodenal epithelium is capable of rapid self-repair. The third level of the gastro-duodenal mucosal barrier is the mucosal microcirculation. Mucosal blood flow is critical for damage limitation and repair facilitation. The tight junction protein occludin plays an important role in the barrier function of the mucosa and acts to effectively prevent the inverse dispersion of hydrogen ions into the gastro-duodenal mucus. No significant difference was identified in the expression level of occludin between the low and high bleeding risk groups in the present study.

NO is a highly reactive molecule that is produced by NOS. It plays a fundamental role in maintaining normal vasomotor tone (15). As mentioned previously in this discussion, mucosal blood flow is an important defensive factor that protects the gastric mucosa. The level of NOS expression in the gastric mucous membrane epithelial cells may, to a certain extent, reflect gastric mucosal blood volume. EGF is a crucial factor for healing in PUD. EGF and EGFR protein expression is involved in the re-epithelialization and peptic ulcer healing process, which is associated with the proliferation, differentiation and migration of gastric mucous surface epithelial cells (16). The histological results of this study showed that the expression levels of the NOS, EGF and EGFR proteins were not significantly different between the high and low bleeding risk groups in the extreme hot or cold climates. Despite this finding, when the associations between the peptic ulcer-related factors and climate were studied, it was found that the expression of occludin, NOS and EGFR was significantly lower in the extreme cold climate than that in the extreme hot climate. This result may explain why the incidence of peptic ulcers increases in the winter and decreases in the summer, exhibiting a clear seasonal pattern. However, it is likely that the seasonal pattern of PUB is not associated with the expression of the NOS, EGF and EGFR proteins in the gastric mucous membrane epithelial cells.

HSPs are a set of proteins whose expression is induced by heat shock and a number of other stresses. In recent years, HSPs have been implicated in gastro-duodenal defense mechanisms at the intracellular level. HSP70 is a type of HSP induced by molecular chaperones, and it is involved in various biological activities, such as rescue from apoptosis, protection from cytotoxic damage, e.g. from NSAIDs or *H. pylori* infection, and facilitation of ulcer healing (17,18). The present results demonstrated that the expression of HSP70 in the high bleeding risk group was significantly lower than that in the low bleeding risk group in the extreme cold climate; however, the difference was not significant in the extreme hot climate. In addition, the expression of HSP70 was significantly lower in the extreme cold climate than that in the extreme hot climate. The present findings suggest that low expression of HSP70 in the gastric mucosa may play a key role in the mechanism of PUB in extreme cold climates.

Mucus is a major gastric mucosal defensive factor that functions at the first level of the gastro-duodenal defense system. In the present study it was observed that the gastric mucus thickness in the high bleeding risk group was significantly lower than that in the low bleeding risk group in the extreme cold climate. This reduction in thickness may weaken the gastro-duodenal mucosal defensive mechanism.

Previous studies have demonstrated that PUD has a seasonal pattern, but it has been unclear whether climatic factors also influence PUB (19,20). The results of the present study indicate that PUB induced by extreme cold climate may be associated with low expression of HSP70 and a reduction in gastric mucus thickness. There is substantial evidence that HSP70 plays an important role in the mechanisms of PUB (21). We hypothesize that extreme cold climate disrupts the barrier factors of the gastric mucosa, thereby permitting aggressive factors to damage the gastro-duodenal epithelium, which may progress to PUB. In the present study, the expression levels of the NOS, EGF and EGFR proteins were not significantly different between the high and low bleeding risk groups in the extreme climates; however, a seasonal fluctuation in PUB was observed. Although the rate of *H. pylori* infection between the high and low bleeding risk groups was statistically insignificant in this study, *H. pylori* infection is the most common pathogen associated with PUD and PUB. In conclusion, the decrease in barrier factors and increase in damage in the extreme cold climate may explain the pathogenesis of PUB in extreme cold conditions. Therefore, enhancing the gastro-duodenal mucosal barrier system could aid in preventing PUB in extreme cold climates.

## Figures and Tables

**Table I:** Patient demographics according to bleeding risk group and extreme climate conditions.

	High bleeding risk group			Low bleeding risk group		
		
Climate condition	Male (n)	Female (n)	Average age (years)	Male (n)	Female (n)	Average age (years)
Hot, >30°C	23	14	40.51±15.73	23	14	41.97±14.92
Cold, <10°C	31	20	40.24±13.33	31	20	41.59±11.66

Average age is presented as the mean ± standard deviation.

**Table II:** pH value and *Helicobacter pylori* infection rate of the gastric antrum in the different groups.

Group	pH value	*H. pylori* infection rate (%)
Extreme hot		
High bleeding risk	1.93±1.04^a^	75.68
Low bleeding risk	2.05±1.27	83.78
Extreme cold		
High bleeding risk	1.00±0.81^a^	86.27
Low bleeding risk	1.35±0.93	74.51

a P<0.05.

pH value is presented as the mean ± standard deviation.

**Table III:** Expression of HSP70 according to patient group.

		HSP70 protein expression (gastric antrum)						HSP70 protein expression (gastric body)					
			
Group	N	− (n)	+ (n)	++ (n)	+++ (n)	PR (%)	P-value	− (n)	+ (n)	++ (n)	+++ (n)	PR (%)	P-value
Extreme hot													
High bleeding risk	37	2	4	11	20	83.8	0.923^a^	1	4	11	21	86.5	<0.001^a^
Low bleeding risk	37	2	5	9	21	81.1	<0.001^b^	0	4	10	23	89.1	<0.001^b^
Extreme cold													
High bleeding risk	51	25	15	3	8	21.6	<0.001^c^	11	31	6	3	17.6	<0.001^c^
Low bleeding risk	51	8	18	20	5	74.5	0.002^d^	5	24	16	6	43.1	0.005^d^

P<0.05 was considered statistically significant.

a High bleeding risk group versus low bleeding risk group in the extreme hot climate;

b Extreme hot climate versus extreme cold climate for the low bleeding risk group;

c Extreme hot climate versus extreme cold climate for the high bleeding risk group;

d High bleeding risk group versus low bleeding risk group in the extreme cold climate.

PR, positive rate; HSP70, heat shock protein 70.
